# The Effects of Magnesium Supplementation on Subjective Anxiety and Stress—A Systematic Review

**DOI:** 10.3390/nu9050429

**Published:** 2017-04-26

**Authors:** Neil Bernard Boyle, Clare Lawton, Louise Dye

**Affiliations:** School of Psychology, University of Leeds, Leeds LS2 9JT, UK; c.l.lawton@leeds.ac.uk (C.L.); l.dye@leeds.ac.uk (L.D.)

**Keywords:** magnesium, anxiety, stress, intervention

## Abstract

Background: Anxiety related conditions are the most common affective disorders present in the general population with a lifetime prevalence of over 15%. Magnesium (Mg) status is associated with subjective anxiety, leading to the proposition that Mg supplementation may attenuate anxiety symptoms. This systematic review examines the available evidence for the efficacy of Mg supplementation in the alleviation of subjective measures of anxiety and stress. Methods: A systematic search of interventions with Mg alone or in combination (up to 5 additional ingredients) was performed in May 2016. Ovid Medline, PsychInfo, Embase, CINAHL and Cochrane databases were searched using equivalent search terms. A grey literature review of relevant sources was also undertaken. Results: 18 studies were included in the review. All reviewed studies recruited samples based upon an existing vulnerability to anxiety: mildly anxious, premenstrual syndrome (PMS), postpartum status, and hypertension. Four/eight studies in anxious samples, four/seven studies in PMS samples, and one/two studies in hypertensive samples reported positive effects of Mg on subjective anxiety outcomes. Mg had no effect on postpartum anxiety. No study administered a validated measure of subjective stress as an outcome. Conclusions: Existing evidence is suggestive of a beneficial effect of Mg on subjective anxiety in anxiety vulnerable samples. However, the quality of the existing evidence is poor. Well-designed randomised controlled trials are required to further confirm the efficacy of Mg supplementation.

## 1. Introduction

Magnesium (Mg) is an essential mineral utilized in the human body, as a cofactor, by in excess of 300 biochemical reactions required to maintain homeostasis [1]. The biological functions of Mg are broad and varied, and include the production of nucleic acids, involvement in all adenosine triphosphate (ATP) fueled reactions, and modulation of any activity mediated by intracellular calcium concentration fluxes (e.g., insulin release, muscle contraction [2]).

Dietary intake of Mg has been shown to be insufficient in Western populations [3,4,5]. Sixty-eight percent of Americans [3] and 72% of middle aged French adults [6] have been shown to consume less than the recommended levels of dietary Mg. This inadequate intake is linked with an array of poor health outcomes including hypertension [7], cardiovascular disease [8], and type II diabetes [9].

Depletion and supplementation studies in animals and humans suggest that Mg may play an important part in the etiology of affective mood disorders. A relationship between Mg and affective depressive states has been established (for reviews see [10,11]). Magnesium plays a key role in the activity of psychoneuroendocrine systems and biological and transduction pathways associated with the pathophysiology of depression. For example, all elements of the limbic–hypothalamus–pituitary–adrenocortical axis are sensitive to the action of Mg [12]. Magnesium has also been demonstrated to suppress hippocampal kindling [13,14], attenuate the release of, and affect adrenocortical sensitivity to, adrenocorticotrophic hormone (ACTH) [15,16], and may influence the access of corticosteroids to the brain at the level of the blood brain barrier via its action on p-glycoprotein [17,18,19].

Experimentally induced hypomagnesemia results in depression like behavior in rodents [20,21,22,23] which is effectively treated by administration of antidepressants [21,23]. An impoverished Mg diet is associated with depression in humans [24]. Low serum and cerebrospinal fluid Mg levels have also been associated with depressive symptomology [25] and suicidality [26]. However, further evidence of a relationship between raised Mg levels and depressive states [27,28,29] suggests the relationship between Mg levels and depression is yet to be fully elucidated.

Further support for a relationship between Mg and affective states comes from evidence of the efficacy of Mg supplementation in the treatment of depression. Magnesium intake reduces depression-related behaviour in mice [30] and is effective as an adjunctive treatment for depression in rodent models [31,32]. In humans, 12 weeks intake of 450 mg of elemental Mg has been shown to be as effective in reducing depression symptoms as a tricyclic antidepressant (Imipramine 50 mg) in depressed hypomagnesic elderly patients with type II diabetes [33]. Further evidence from case studies suggests Mg is an effective adjunctive therapy for treating major depression [34,35]. However, the efficacy of Mg in the treatment of depression symptomology has not been consistently reported [36]. Mood stabilizing effects of Mg supplementation have also been reported in additional clinical samples, including the improvement of clinical signs of mania [37], rapid cycling bipolar disorder [38], and alleviation of affective symptoms associated with chronic fatigue syndrome [39].

Depression is often comorbid with anxiety [40]. Anxiety related conditions are the most common affective disorders present in the general population with a lifetime prevalence of over 15% [41]. The anxiolytic potential of Mg has been demonstrated in rodent models. Naturally and experimentally induced hypomagnesemia elevates anxiety states in mouse models [12,21,42,43]. Blood plasma and brain Mg levels are also significantly correlated with anxiety-related behavioral responses in rodents [44]. Supplementing Mg levels in mice has been demonstrated to reduce the expression of anxiety-related behavior [30,45].

A relationship between Mg status and anxiety is evident in humans. Test anxiety, related to exposure to stressful exam conditions, increases urinary Mg excretion, resulting in a partial reduction of Mg levels [46]. Further, dietary levels of Mg intake have been modestly inversely associated with subjective anxiety in a large community-based adult sample [24]. Magnesium also modulates activity of the hypothalamic pituitary adrenal axis (HPAA) which is a central substrate of the stress response system. Activation of the HPAA instigates adaptive autonomic, neuroendocrine, and behavioral responses to cope with the demands of the stressor; including increasing anxiety. Exposure to stress moderates serum (noise stress; [47]) and intracellular (exam stress; [48]) Mg levels. Magnesium supplementation has also been shown to attenuate the activity of the HPAA, including a reduction in central (ACTH; [15]) and peripheral (cortisol; [49]) endocrine responses of this system. Therefore, Mg may further influence anxiety states via the moderation of the stress response.

A number of potential mechanistic pathways have been described which may account for the relationship between Mg and anxiety. Glutamate is the primary excitatory neurotransmitter in the mammalian brain. Glutamate acts on Ca^2+^ channel coupled N-methyl-D-aspartate (NMDA) ionotropic receptors which have been implicated in anxiety and panic disorders [50]. Magnesium reduces neuronal hyperexcitability by inhibiting NMDA receptor activity [51]. Magnesium is also essential for the activity of mGluRs—G-protein coupled receptors that are widely expressed in the brain [52,53]. The mGluRs receptors play a key modulatory role in glutamatergic activity, secretion and presynaptic release of glutamate, activity of the GABA (γ-aminobutyric acid)ergic system, and regulation of the neuroendocrine system. The action of glutamate on mGluRs receptors has been implicated in responses to fear, anxiety and panic [53]. Magnesium may additionally modulate anxiety via increasing GABAergic availability by decreasing presynaptic glutamate release [54]. GABA is a primary inhibitory transmitters in the CNS that counterbalances the excitatory action of glutamate. An imbalance between GABA and glutamate is associated with neuronal hyperexcitability characteristic of pathological anxiogenesis [55].

Evidence of the association between Mg and anxiety has increased interest in the potential efficacy of Mg intake to attenuate anxiety symptoms. Prevalent pharmaceutical anxiolytic treatments for clinical anxiety (e.g., benzodiazepines) are often characterized by multiple negative side-effects for many patients. Therefore, the identification of new efficacious treatments to alleviate symptoms of anxiety has great utility. This systematic review summarises the current available evidence for the efficacy of Mg supplementation in the alleviation of subjective measures of anxiety. Considering the conceptual and psychoneuroendocrine overlap between anxiety and stress, the review will also examine evidence for potential effects of Mg intake on parameters of subjective stress. A previous systematic review of the effects of nutritional and herbal supplements on anxiety and anxiety-related disorders summarised the findings of three Mg intervention studies [56]. However, this review summarised the literature prior to 2010 and, since searches were limited to only two databases, likely failed to identify all relevant publications. Therefore, this is the first systematic review of the relationship between Mg supplementation and subjective anxiety and stress.

## 2. Materials and Methods

### 2.1. Selection of Studies

The research synthesis was limited to intervention studies in human adult samples (≥18 years old.) that administered a Mg dose in isolation or combined with a maximum of 5 additional ingredients, and reported an outcome measure of subjective anxiety or stress. This included any general subjective measure that included subscales related to stress and anxiety symptomology. Intervention studies examining acute and chronic effects of Mg manipulations were included. Studies examining the effects of Mg depletion (in the absence of an intervention) or increased consumption of diets associated with high Mg content were excluded. Studies reporting effects in individuals with significant health conditions (e.g., cancer, chronic fatigue syndrome) and developmental disorders (e.g., autism) were excluded. Samples recruited on the basis of mild to moderate subjective anxiety, hypertension, or subjective symptoms associated with premenstrual syndrome (PMS), were retained. The efficacy of Mg intake has been examined as a novel and adjunct treatment approach for depression. This literature has been adequately summarised in a number of previous systematic reviews (e.g., [10,11]). Therefore, studies reporting the effects of Mg intake in depressed samples are not reviewed here. Publications were required to be in the English, French or German languages to permit review by authors. Studies failing to report sufficient detail to permit accurate characterisation of the methodological approach were also not included in the review. Minimum reporting requirements were sample size and composition, Mg dose and intervention length, and clearly defined outcome measures of subjective anxiety or stress.

### 2.2. Literature Search

To identify relevant studies, computerised database searches were conducted in May 2016 on OVID MEDLINE (inclusive of records 1946–2016 and non-indexed citations, 2016), PsycINFO (inclusive of records 1806–2016), EMBASE (inclusive of records 1806–2016, CINAHL (inclusive of records 1960–May 2016), and the databases comprised under EBM REVIEWS (inclusive of records 1991–2016). The following search terms were used: ‘Magnesium$’; OR ‘Epsom’; OR ‘Mg citrate’; OR ‘Mg oxide’; OR ‘Mg sulphate’; OR ‘Mg lysinate’; OR ‘Mg glycinate’; OR ‘Mg bicarbonate’; OR ‘Mg carbonate’; OR ‘Mg chloride’; OR ‘Mg hydroxide’; ‘Mg phosphate’; OR ‘Mg ascorbate’; OR ‘Mg aspartate’; OR ‘Mg fumarate’; OR ‘Mg gluconate’; OR ‘Mg glutamate’; OR ‘Mg lactate’; OR ‘Mg malate’; OR ‘Mg pidolate’; OR ‘Mg orotate’; OR ‘Mg taurate’ AND ‘Stress$’; OR ‘Strain’; OR ‘Tension’; OR ‘Cortisol’; OR ‘Anxi$’; OR ‘Worry’; OR ‘Mood’. For searches of OVID MEDLINE, PSYCH INFO, EMBASE the search field (tw—text word) was applied to all search terms. The search field (tx—full text) was applied for searches of CINAHL and EBM REVIEWS. The additional filter ‘Human’ was added to all searches where supported. The reference lists of existing reviews and identified articles were hand searched to supplement the electronic searches.

**Figure 1:**
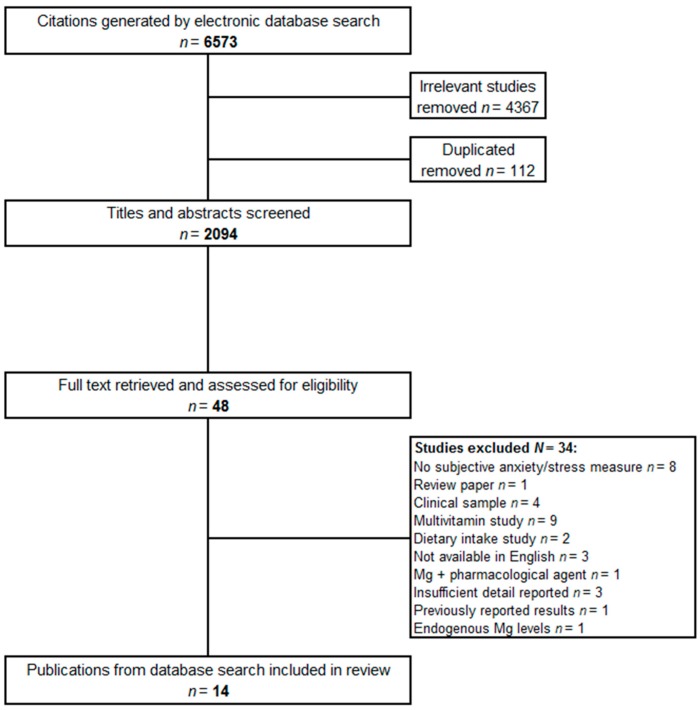
Electronic database study selection summary.

The database searches returned at total of 6573 articles. Publication titles were reviewed to remove patently irrelevant and duplicate papers, leaving a total of 2094 articles selected for abstract review. The full text versions of 48 articles were retrieved and examined for eligibility. A further 34 articles were excluded (reasons for exclusion are shown in Figure 1) leaving 14 studies that met the review criteria.

**Figure 2:**
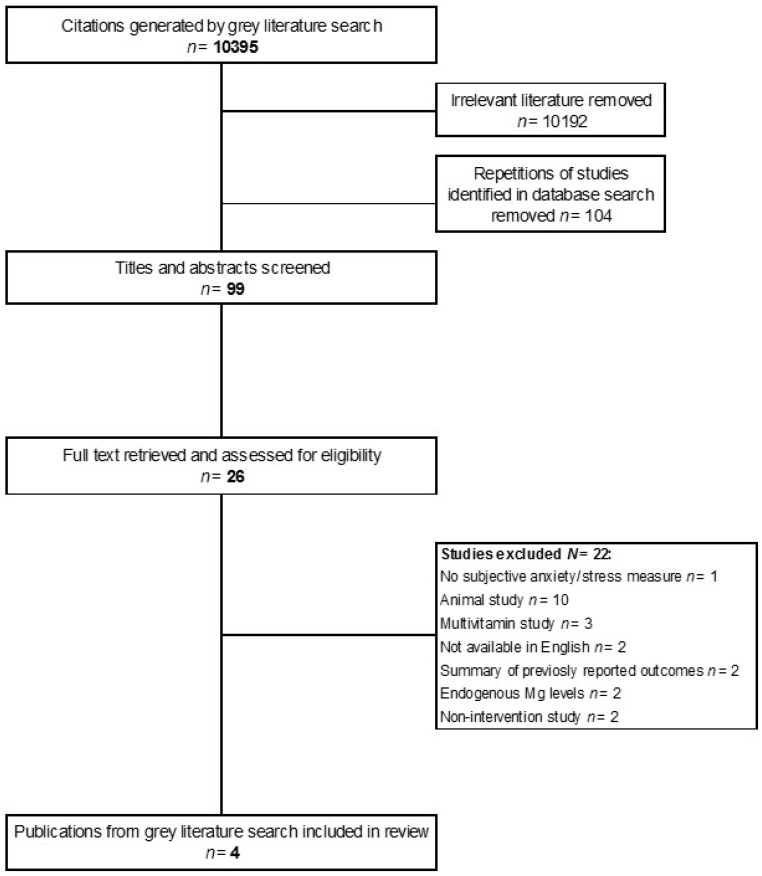
Grey literature search study selection summary.

A grey literature search was also undertaken (September 2016) using grey literature search engines, a google scholar search, and targeted websites using the search terms ‘magnesium’ AND ‘anxiety’ OR ‘stress’. A full list of employed grey literature resources is shown in Appendix A. A request for unpublished data was also published in Magnesium Research [57] and circulated on Researchgate.net. A total of 10,395 citations were screened for relevance. A summary of the grey literature search is shown in Figure 2. The search returned 4 relevant studies which were included in the review. All 4 studies were unpublished in full form at the time of the search. One study is cited in a European Food Safety Authority (EFSA) scientific opinion claim on Mg supplementation [58]. Three internal studies conducted by Sanofi S.A were included in the review. A conference abstract of the Rouillon et al. study was published in 1995 [59]. Two studies by Caillard [60,61] have not been published. Full data from these studies were provided by Sanofi, France. A short summary of these data has been published previously [62].

### 2.3. Data Extraction

The following information was extracted from the reviewed studies:

***Study Design*:** the experimental designs employed in each study were coded as randomised controlled trial (RCT); parallel groups (P); randomised crossover (R-Cross); and non-randomised crossover (NR-Cross). ***Condition:*** all of the reviewed studies recruited samples based upon a specific inclusion criterion; namely, mild to moderate subjective anxiety, premenstrual syndrome (PMS), <48 h postpartum, and mild hypertension. The specific inclusion criterion, and the measure/method employed to identify suitable samples, were extracted. ***Sample Characteristics:*** the sample size and composition (male (M), female (F), mixed (*n*M:*n*F)), and age (*mean*, *SD* and range reported if available). ***Treatment:*** the form (when reported) and dose of Mg administered and additional ingredients were reported in milligrams (mg). ***Control:*** the type of control, if employed, administered (e.g., placebo, active verum). ***Duration:*** the length of time the Mg intervention was administered. ***Results:*** a summary of the analyses including means and SDs (if reported) of any significant findings. ***Effect Summary:*** the reported effects of Mg administration were summarised as positive effect (+), no effect (×), negative effect (−), and (?) if there exists some doubt regards the reported outcome.

## 3. Results and Discussion

From the 18 studies meeting the review inclusion criteria, ten recruited mixed sex samples. Eight studies that examined the effects of Mg intake on PMS symptomology, and one study assessing postpartum anxiety, recruited female samples. The Mg doses administered ranged from 46.4–600 mg. Only one study adjusted a Mg dose relative to body weight (intravenous Mg sulphate infusion 0.1 mmol/kg; [63]) and one study considered potential dose response effects (administering 200, 350, and 500 mg doses; [64]). Magnesium lactate was the most commonly administered Mg form (*n* = 5 studies) followed by Mg oxide (*n* = 4). Seven studies combined Mg with vitamin B_6_ and two studies administered Mg with extract of Hawthorn.

All the reviewed studies recruited samples based upon specific anxiety ‘vulnerability’ criteria. Eight studies recruited individuals reporting mild to moderate subjective anxiety; the majority of which (6/8 studies) applied a score range of 10–30 on the Hamilton Anxiety Scale (HAM-A) [65] as an eligibility criterion. Seven studies recruited women reporting mild to moderate PMS symptoms. Eligibility was determined during menstrual cycle(s) prior to study entry using the Moos Menstrual Distress Questionnaire [66], menstrual health questionnaires [67], or subjective report. One study examined the effects of Mg intake on postpartum anxiety ratings [68]. Two studies recruited participants with mild hypertension, defined as diastolic blood pressure (BP) 85–100 mmHg [69], or diastolic and systolic BP > 90 mmHg and 140 mmHg respectively [70].

No study administered a validated measure of subjective stress as an outcome. A number of general well-being measures were employed that included stress-related subscales (e.g., tension, concerns about the future). However, these offer insufficient evidence to form any valid judgement on the efficacy of Mg on subjective measures of stress. Validated measures of subjective anxiety (e.g., HAM-A; Spielberger State Trait Anxiety Inventory (STAI) [71]), and menstrual symptom and general well-being measures which included subscales specifically related to subjective anxiety (e.g., Moos Menstrual Distress Questionnaire (MDQ) [66]) were employed. Evidence of the effect of Mg intake on subjective anxiety outcomes is reviewed separately for each anxiety vulnerability subgroup type.

### 3.1. Mild Anxiety

A summary of studies examining the effects of Mg intake in anxious samples is shown in Table 1. Three of the eight studies which recruited samples based upon pre-existing levels of mild subjective anxiety reported positive effects of Mg supplementation on anxiety outcomes. Two unpublished RCTs compared six weeks administration of 192 mg Mg lactate + vitamin B_6_ (20 mg) vs. placebo. A greater change from baseline reduction in the HAM-A ratings after 21 days of Mg + vitamin B_6_ intake compared to the placebo was reported (*p* < 0.03; [60]). However, this superiority of Mg over placebo was not maintained after 42 days. A RCT, identical in design and dose, in a larger sample focussed on the somatic features of anxiety. This reported significantly lowered somatic anxiety symptoms on the HAM-A scale after 21 (*p* < 0.004) and 42 (*p* < 0.02) days treatment with Mg + vitamin B_6_ vs. placebo ([61]). Whilst both studies demonstrated a greater reduction in anxiety after Mg + vitamin B_6_ compared to placebo, a sizeable placebo effect was also evident.

Hanus et al. [72] reported positive effects of 12 weeks intake of 75 mg Mg combined with Hawthorn (75 mg) and California poppy (20 mg) extracts vs. a placebo in individuals reporting mild anxiety or symptoms of general anxiety disorder. Consistent positive effects on three anxiety outcome measures were reported. A significant decrease from baseline in HAM-A total anxiety score was demonstrated in both Mg and placebo conditions after 90 days intake. However, the effect was significantly greater in the Mg treatment group (*p* = 0.005). A comparable pattern of effect was shown for HAM-A somatic anxiety (*p* = 0.054). Both Mg treatment and placebo also demonstrated a significant reduction from baseline after 90 days on a subjective anxiety visual analogue scale (VAS). The reduction was again greater in the treatment group (*p* = 0.005). Finally, a physician global impression rating, a subjective efficacy ratio rating of the benefit vs. risk of a treatment, was significantly higher for Mg treatment vs. placebo (*p* = 0.0018).

Cazaubiel and Desor [58] reported a significant reduction in the anxiety subscale of the Hospital Anxiety and Depression Scale (HADS; [73]) in a mildly anxious sample following 4 weeks intake of a fermented milk drink combined with 48 mg Mg. However, this finding can be considered unreliable as it reflects a post hoc analysis on restricted data (post hoc re-categorisation of ‘mild stress’) in a reduced subsample (*n* = 15).

Three studies compared Mg + vitamin B_6_ with a pharmaceutical anxiolytic as a positive verum. Two studies compared six weeks intake of 300 mg Mg lactate + vitamin B_6_ (20 mg) vs. 3 mg [74] or 2 mg [75] of Lorazepam vs. Lorazepam combined with 300 mg Mg + vitamin B_6_. A reduction in HAM-A rating was evident in all treatments but no significant differences between the conditions were found. Similarly, despite a reduction in ratings in both conditions, Rouillon, Lejoyeux, & Martineau [59] found no significant difference between 192 mg Mg lactate + vitamin B_6_ vs. 40 mg Buspirone on HAM-A ratings after 6 weeks intake. An initial 7 day placebo washout period was employed prior to full study participation in this study to remove participants that exhibited sizeable placebo effects (≥50% improvement in total HAM-A score). Comparable efficacy with pharmaceutical anxiolytics may be considered evidence to support the positive effect of Mg on subjective anxiety. However, the lack of placebo control in these studies should be noted, particularly in the light of the significant placebo response seen in the 3 studies in which a placebo was administered. Further, the addition of a positive verum in studies that did administer a placebo control, which would have permitted both a non-active, and a proven, active comparison, would have provided a more distinct measure of the efficacy of Mg.

The final study reporting no effects of Mg compared pre-exam test anxiety in university students after 5 days intake of 300 mg Mg citrate vs. placebo [76]. The authors categorised participants into four anxiety groups based on subjective ratings prior to the intervention, ranging from normal to very high subjective anxiety. No differences between anxiety ratings (STAI) on the eve of the exam were found between conditions or as a function of anxiety group categorisation. This lack of effect may be due to contextual differences in the form of anxiety examined. Whilst positive evidence of the anxiolytic effects of Mg has been shown in chronically anxious samples (i.e., those demonstrating moderate anxiety scores on the HAM-A), Gendle et al. [76] examined the effects of Mg on responses to an acute, anxiety-provoking situation-specific context. Whilst the authors did take into account pre-existing levels of anxiety in the sample, this was ascertained by the Westside Test Anxiety Scale [77] which is a short measure specifically designed to assess exam-specific, not clinical, anxiety and was used as a covariate in the analysis rather than to select an anxiety vulnerable sample. Therefore, both the context and sample differ from the other studies reviewed which recruited chronically anxious individuals using a clinical measure; the HAM-A.

Examining the efficacy of Mg to reduce subjective anxiety in anxious individuals is a valid approach. The positive effects of nutritional interventions are often demonstrated in those with specific pre-existing vulnerabilities (e.g., low socio-economic status [78]; low IQ [79]; high neuroticism [80]). However, six out of eight studies examining the effects of Mg intake in anxious samples employed the HAM-A both as an inclusion criterion and primary outcome variable. This practice has the effect of constraining the variance of responses at inclusion, increasing the likelihood of regression to the mean post-intervention [81] and may therefore mask some of the true effect if it exists in the population [82]. The employment of a measure to identify anxious samples that is distinct from the subjective anxiety outcome measure is preferable.

#### Summary of Effects of Mg in Anxious Samples

Findings to date offer modest support that Mg intake confers benefits for individuals with pre-existing mild to moderate levels of anxiety. Four out of eight studies reported positive effects of Mg intake on anxiety outcomes. However, three studies are unpublished and one of these reports unreliable post hoc analyses in a significantly reduced subsample. Those studies which reported positive effects all administered Mg in combination with additional ingredients (e.g., vitamin B_6_, extract of Hawthorn and California poppy). None of the studies examined the effects of the included ingredients in isolation. Therefore, it is not possible to distinguish the relative contribution of each ingredient or confirm whether the positive effects observed are additive or synergistic. Three studies reported comparable efficacy between Mg and pharmaceutical anxiolytics. Whilst the strength of this evidence is diminished by a lack of a placebo comparator, it is indicative of the potential positive efficacy of Mg. Non-inferiority in treatment effect of Mg supplementation compared to a proven, efficacious anxiolytic (e.g., Buspirone [83,84]) is suggestive of a promising role for Mg supplementation in the alleviation of subjective anxiety symptomology; especially considering the negative side effects associated with pharmaceutical anxiolytic intake and comparative safety of Mg supplementation. Mg All of the studies which included a placebo demonstrated significant placebo effects. Whilst positive effects of Mg were reported to be in excess of the effects of placebo, significant placebo effects suggest that any intervention in anxiety vulnerable samples may result in an amelioration of subjective anxiety complaints. The inclusion of an appropriate placebo to evaluate the effects of Mg interventions is therefore critical.

### 3.2. Premenstrual Syndrome

A summary of studies examining the effects of Mg intake in samples reporting PMS symptoms is shown in Table 2. Four of the seven studies recruiting samples based upon pre-existing PMS symptoms reported positive effects of Mg supplementation on anxiety outcomes. However, this positive evidence is undermined by a number of methodological limitations. De Souza et al. [67] administered 200 mg Mg oxide alone and combined with vitamin B_6_ (50 mg) vs. vitamin B_6_ (50 mg) alone vs. a placebo for five consecutive menstrual cycles in a crossover manner. A significant reduction of anxiety-related premenstrual symptoms (nervous tension, mood swings, irritability, and anxiety) vs. baseline and placebo was reported after 200 mg Mg oxide + vitamin B_6_ (*p* = 0.04). However, no overall treatment effects were found; the effect reported is the result of a priori planned treatment contrasts. 

Quaranta et al. [85] administered 250 mg Mg in a modified release capsule for three menstrual cycles. Treatment significantly reduced total score on the Moos MDQ (including nervous tension and anxiety subscales; *p* < 0.001), and on an anxiety subscale of a monthly PMS symptom diary (*p* < 0.001). However, these effects were relative to screening visit and baseline scores respectively. This study failed to administer any form of control or placebo. This is a methodological concern given evidence of the significant placebo effects previously discussed and demonstrated in PMS [86]. Indeed, Fathizadeh et al. [87] reported that 250 mg Mg, alone and combined with vitamin B_6_, and a placebo, all resulted in a significant reduction in subjective PMS symptoms. Whilst the authors report that 250 mg Mg + vitamin B_6_ resulted in the greatest symptom amelioration (*p* < 0.05), these findings emphasise the robustness of the placebo effect in PMS samples and the need to evaluate active treatments against placebo treatments. The authors also analysed the effects of treatments on specific subjective PMS symptom subscales and reported a main effect of treatment on anxiety-related symptomology. However, appropriate follow up tests were not performed to distinguish between the treatment groups.

The final study reporting positive effects of Mg administered 360 mg pyrrolidone carboxylic acid Mg vs. placebo [88]. Magnesium intake significantly reduced subjective premenstrual negative affect symptoms on the Moos MDQ, which includes the symptoms anxiety and tension [66]. This effect of Mg was shown in a placebo/treatment crossover condition (2 months placebo intake vs. 2 months Mg intake; *p* < 0.05), and after 2 and 4 months intake (vs. baseline) in a Mg treatment condition (*p* < 0.05).

Three studies reported no effects of Mg intake on anxiety-related PMS symptoms. Walker et al. [89] found no effects of 2 months administration of 200 mg Mg oxide on PMS symptomology. A further study by this group examined potential Mg dose-response effects by administering 200, 350, and 500 mg Mg oxide in a placebo controlled crossover trial [64]. Placebo intake (1305 mg sorbitol) significantly reduced subjective total and anxiety-related PMS symptoms after 2 months compared to all doses of Mg (*p* < 0.001). The authors suggest the positive effects of sorbitol may be due to the raising of depleted intracellular sorbitol concentrations caused by hypoglycaemia, which is a suggested—though unconfirmed—symptom of PMS. Therefore, the effect of sorbitol may be specific to the PMS sample. Indeed, the authors additionally demonstrated that sorbitol reduced urinary Mg excretion in PMS (vs. baseline and Mg treatments), but not asymptomatic controls (vs. baseline and cellulose placebo). The dose and duration of intake may also be relevant since effects were not evident after one month, and the Mg treatments contained smaller doses of sorbitol (Mg 200 mg = 1050 mg; 350 mg = 830 mg; 500 mg = 717 mg sorbitol). It is not possible to discern the extent to which this finding is of relevance to other PMS studies reporting placebo effects as the nature of the placebo has rarely been detailed. Only Walker et al. [89] report the form of placebo administered (cellulose). Therefore, the potential impact of placebo content on the effects observed in PMS symptom samples is not known.

In a methodologically flawed study, Khine et al. [63] initially adopted a parallel groups design comparing women reporting PMS complaints or meeting the criteria for premenstrual dysphoric disorder (PMDD) with non PMS controls. The authors administered 0.1 mmol/kg body mass of Mg sulphate via intravenous infusion over four hours. The acute subjective effects of Mg infusion were assessed 24 h later by the STAI, the Premenstrual Tension Scale [90] and a PMS symptom VAS. The study design was altered mid-testing after improved VAS mood ratings were reported in the PMS/PMDD participants only (*n* = 6). The Mg and a placebo infusion were subsequently administered to 10 more PMS/PMDD women in a crossover manner. No significant differences between Mg and placebo were demonstrated on any subjective outcomes in this subsequently combined sample. Moreover, any outcomes are crucially compromised due to the decision to alter the study design based upon emerging data.

The heterogeneity in the evidence for the efficacy of Mg in treatment of anxiety-related PMS symptoms may be explained by the divergent methods employed to characterise PMS samples. For example, four studies [64,67,85,89] employed retrospective assessment of PMS symptoms over the previous month and/or at baseline. The reliance on retrospective diagnosis has been criticized [91] since these often result in inflated estimates of symptom severity [92]. Only Facchinetti et al. [88] report the assessment of daily PMS symptoms in the 2 months prior to study commencement to identify eligibility (according to DSM-IIIR criteria). Khine et al. [63] and Fathizadeh et al. [87] also collected daily symptom records in the months (3 and 2 months respectively) prior to study commencement. However, not enough detail is reported to determine by which criteria participant eligibility was ascertained. Therefore, it is not easy to assess the equivalency of PMS symptom severity across the samples. This is problematic if, for example, the potential functional effects of Mg supplementation operate as a function of PMS symptom severity (e.g., attenuating symptoms in mild or very severe cases). A more consistent approach to assessing PMS symptomology prior to inclusion may reduce some of the variability evident in the existing literature.

#### Summary of Effects of Mg in PMS Samples

The findings to date suggest a potential positive role for Mg supplementation on subjective anxiety in women reporting PMS symptoms. Positive effects of Mg were reported both in isolation and when combined with vitamin B_6_. Studies reporting positive effects of Mg combined with vitamin B_6_ demonstrated effects superior to those of Mg administered alone. However, evidence of the effects of Mg intake on subjective PMS related anxiety are undermined by a number of methodological issues. A lack, or inappropriate application, of a placebo control, and design and analysis issues all contribute to the ability to draw clear conclusions as regards this problem. Careful selection of an appropriate placebo control for samples of this nature (sub-clinical complaints) is also highlighted by the apparent specific functional effects of sorbitol on women with PMS. A more consistent approach to diagnosis (preferably using DSM-IV criteria and characterising PMS samples is required to permit better assessment of the equivalency of PMS symptom severity and response to treatment between studies.

### 3.3. Postpartum Anxiety

One study examined the capacity of Mg intake to ameliorate postpartum (≤48 h) anxiety in a healthy sample (Table 3). No significant effects on subjective anxiety rating (STAI) in the eight weeks following child birth were recorded following daily supplementation with 64.4 mg Mg (vs. placebo and zinc; [68]).

#### Summary of Effects of Mg in Postpartum

There is currently no evidence to support the efficacy of Mg intake in the reduction of postpartum subjective anxiety.

### 3.4. Mild Hypertension

A summary of studies examining the effects of Mg intake in mild hypertensive samples is shown in Table 4. Both studies employed general subjective measures of quality of life (QoL) and well-being which comprised subscales related to stress and anxiety. Therefore, the findings are considered to contribute only modest evidence to support the examination of the efficacy of Mg intake on anxiety/stress. Borrello et al. [70] administered 200 mg Mg oxide vs. placebo for 12 weeks in a hypertensive sample. Magnesium intake resulted in significantly higher QoL ratings (inclusive of scales measuring subjective emotional behaviour and concerns about the future) vs. placebo and baseline at 12 weeks (*p* < 0.05). Conversely, Walker et al. [69] found no effects of 10 weeks intake of 600 mg Mg chelate on a subjective well-being measure (comprising an anxiety subscale) when administered in isolation or in combination with Hawthorn extract (500 mg).

#### Summary of Effects of Mg in Hypertensive Samples

Evidence of specific anxiety/stress reducing effects of Mg intake in hypertensive individuals is weak due to the inconsistency of evidence and failure to employ specific subjective anxiety/stress outcome measures. However, the capacity for Mg intake to affect subjective indices of mood (QoL) suggests further examination of the efficacy of Mg in hypertensive samples is warranted. Evaluating the efficacy of Mg on subjective anxiety in samples with clinical conditions is complicated by the underlying clinical complaint. For example, an improvement in subjective anxiety may be as a result of an improvement in the clinical symptomology rather than a direct effect of Mg on anxiety. However, this is contradicted by the current available evidence as both studies in hypertensive samples demonstrated Mg reduced blood pressure responses but did not affect subjective anxiety levels.

### 3.5. Moderating Variables

#### 3.5.1. Dosage and Differential Bioavailability of Mg Forms

No clear dose effect of Mg emerges from the reviewed studies. Positive effects of Mg intake on subjective anxiety outcomes are reported with both low (75 mg [72]) and higher (360 mg [88]) Mg doses. One study that systematically examined the potential dosing effects of Mg (administering 200, 350, and 500 mg) reported no effects of any dose [64]. Examination of the effect of Mg dose is further complicated by a number of the studies reporting positive outcomes combining Mg with additional ingredients (e.g., Hawthorn extract [72]). Therefore, it is difficult to assess if it is Mg administered at a particular dose that is efficacious, or the additional ingredients acting in isolation or synergistically with Mg.

An additional factor that needs to be acknowledged is the variable bioavailability of different Mg forms. Magnesium chloride, sulphate, citrate, lactate, malate, glycinate and taurinate are highly biologically available whilst Mg oxide appears to be significantly less bioavailable [93,94,95]. However, there is no consistent moderating effect of Mg form on reported anxiety outcomes. Four studies administered Mg oxide [64,67,70,89]. Two studies reported positive effects of Mg oxide intake, however, positive effects were observed only when combined with vitamin B_6_ [67] and on a subjective general well-being questionnaire including anxiety-related factors rather than specific measures of anxiety [70]. Two of five studies administering Mg lactate reported positive effects of this Mg form. One study reported pyrrolidone carboxylic acid reduced subjective negative affect. Conversely, no effects of citrate, sulphate (intravenous) or amino acid chelate Mg forms have been demonstrated. Therefore, whilst the bioavailability of Mg forms should be considered when planning an intervention, the current available evidence is not sufficient to determine the relative efficacy of specific Mg forms in the attenuation of subjective anxiety outcomes.

#### 3.5.2. Duration of Intake

The majority of the existing evidence of the positive effects is from studies administering Mg for 6–12 weeks. However, this is also true for studies reporting no effects of Mg. Hence, intervention length may not be the principal moderating variable contributing to the heterogeneous effects. There is a paucity of research assessing the acute effects of Mg intake in humans. Gendle et al. [76] reported no effects after 5 days but the sample and anxiety context were markedly different to those sub-clinical and chronically anxious samples recruited in studies reporting positive effects of Mg. Khine et al. [63] reported no effect of an acute intravenous Mg dose but methodological flaws in this study undermine interpretation of the findings.

#### 3.5.3. Mg Status

The majority of studies summarised cite the observed relationship between Mg depletion and affective states as a rationale to hypothesise a potential positive effect of Mg supplementation on subjective anxiety. The exclusive selection of anxiety vulnerable samples (e.g., moderately anxious, PMS symptomology) is based on an assumption that the positive effects of Mg supplementation are more likely observed in those that are compromised or depleted in some way. However, none of the reported studies specifically recruited samples depleted in Mg to assess the effect of Mg intake. A number of studies measured urinary and/or serum Mg status at baseline and over the course/at the completion of the study. These measures were recorded only to confirm the equivalence of treatment groups at baseline and increased Mg bioavailability in Mg conditions or protocol compliance. No attempt was made to incorporate basal Mg status in the statistical analyses of Mg outcomes.

## 4. Conclusions and Research Recommendations

In conclusion, there is suggestive but inconclusive evidence for a beneficial effect of Mg supplementation in mild anxiety. Similarly the evidence from studies of women who complain of premenstrual symptoms also suggests that Mg could confer benefits. In both cases this is based on a reasonable number of studies which have used appropriate measures of symptoms. However, the weaknesses in the designs highlighted and the substantial placebo response noted in most studies preclude strong recommendations for Mg as a treatment option at this stage. The evidence for Mg in hypertension is based on only two studies, both of which do not measure specific symptoms but generic quality of life indices which are unlikely to detect changes in underlying specific symptoms.

The quality of studies was generally poor. Studies that included a placebo condition often failed to evaluate effects appropriately. Studies were marred by inappropriate selection of samples, failure to confirm diagnosis, lack of placebo controls, and weak statistical analysis. It is clear therefore that well-designed randomised controlled trials are required. Such trials should include careful screening of samples and confirmation of the presence of anxiety at levels where a treatment effect would be noticeable (e.g., mild, moderate) on measures with sufficient range. The specific examination of Mg efficacy in individuals with lowered Mg resources is also recommended considering the evidence of the relationship between the depleted state and affective pathologies. The inclusion of a placebo control (with documented content) is crucial as is appropriate power to detect treatment effects and an appropriate statistical analysis strategy, which includes consideration of baseline symptoms as a covariate rather than relative to screening along with planned comparisons against the placebo treatment. Longer term studies should also consider the inclusion of a placebo run-in, whilst acknowledging that placebo response can be quite prolonged in studies of subjective symptoms such as anxiety or PMS. The lack of significant differences between proven anxiolytic pharmaceuticals and Mg intake in the alleviation of subjective stress ratings suggests study designs may also benefit from the inclusion of a positive verum. This would permit a fair assessment of the efficacy of Mg.

The effects of Mg on clinical affective disorders and experimental studies of anxiety in animal models provide a clear rationale to propose that Mg supplementation will have a beneficial effect on mild/moderate anxiety. There is also sufficient potential mechanistic pathways via which Mg could modulate affective states. It is the quality of the available evidence rather than the absence of a potential mechanism which has hindered convincing demonstration of such effects.

The potential effect of Mg in attenuating psychological response to stress merits further investigation since stress is a ubiquitous feature of modern lives. The modulation of the HPA axis by Mg, which has been demonstrated to reduce central (ACTH; [15]) and peripheral (cortisol; [49]) endocrine responses, suggests that behavioural effects of stress exposure such as anxiety could be attenuated by Mg supplementation.

## Figures and Tables

**Table 1:** Summary of studies reporting the effects of Mg on subjective anxiety/stress in mild to moderately anxious individuals.

Author	Study Design	Condition	Sample (*N*)	Sex	Age (year)	Treatment (s)	Control	Duration	Outcome Measure	Results	Effect Summary
Bourgeois et al. [74]	RCT	Mild anxiety (Hamilton Anxiety Scale score 10–30)	*N* = 81 (*n* = 27 per condition)	20M:61F	18–65	(i) Mg 300 mg as lactate + vit B_6_ 750 mg; (ii) Lorazepam 3 mg; (iii) (i) + (ii) combined	Lorazepam 3 mg (positive verum)	6 weeks	Hamilton Anxiety Scale	Reduced anxiety scores in all treatments. No significant differences between treatments.	x *
Scharbach [75]	RCT	Mild anxiety (Hamilton Anxiety Scale score 15–30)	*N* = 133 (Treatments (i) *n* = 44; (ii) *n* = 46; (ii) *n* = 43)	32M:109F	18–65	(i) Mg 300 mg as lactate + vit B6 750 mg; (ii) Lorazepam 2 mg; (iii) (i) + (ii) combined	Lorazepam 2 mg (positive verum)	6 weeks	Hamilton Anxiety Scale	Reduced anxiety scores in all treatments. No significant differences between treatments.	x *
Caillard [60]	RCT	Mild anxiety/general anxiety disorder (Hamilton Anxiety Scale score 15–30 & general anxiety disorder (DSM III criteria))	*N* = 93	25M:68F	*x* = 41 (SD = 12; 18–65)	Mg 192 mg as lactate + vit B_6_ 20 mg	Placebo	6 weeks	Hamilton Anxiety Scale	Significant change from baseline (Total score) between groups at Day 21 (Mg + vit B_6_: *x* = 12.1 (SD = 6.0); placebo: *x* = 15.5 (SD = 5.8)) vs. Day 0 (Mg + vit B_6_: *x* = 21.0 (SD = 4.5); placebo: *x* = 22.6 (SD = 4.4); *p* < .03). No significant differences between Day 0 & Day 42.	+
Rouillon et al., [59]	RCT	Mild anxiety/general anxiety disorder Hamilton Anxiety Scale score 15–30 & general anxiety disorder (DSM III-R criteria))	*N* = 99 (Mg *n* = 51; Buspirone *n* = 48)	38M:61F	*x* = 37.7 (SD = 10.7; 19–65)	Mg 192 mg as lactate + vit B_6_ 20 mg	Buspirone 40 mg (positive verum)	6 weeks	Hamilton Anxiety Scale	Decrease in anxiety scores in both treatment groups across intake. No significant difference between the efficacy of Mg + vit B_6_ & Buspirone.	x *
Caillard [61]	RCT	Symptoms of functional impairment associated with anxiety or a somatic disorder (Hamilton Anxiety Scale ^1^; Raskin depression scale < 7; COVI anxiety scale = 7)	*N* = 103	26M:77F	*x* = 37 (18–65)	Mg 192 mg as lactate + vit B_6_ 20 mg	Placebo	6 weeks	Hamilton Anxiety Scale (somatic score)	Significantly lower somatic anxiety rating after treatment at Day 21 (*x* = 8.4 (SD = 3.8); *p* = 0.004) & Day 42 (*x* = 6.5 (SD = 3.0); p = 0.02) vs. placebo (Day 21: *x* = 9.9 (SD = 2.9); Day 42: *x* = 7.8 (SD = 3.6)).	+
Hanus et al. [72]	RCT	Mild anxiety/general anxiety order (Hamilton Anxiety Scale score 16–28 & somatic score ≥ 50% total score; & general anxiety disorder) DSM-III-R))	*N* = 264 (Treatment *n* = 130; Placebo *n* = 134)	26M:213F	Placebo: *x* = 44.5 (18–82); Treatment: *x* = 44.8 (19–81)	Hawthorn extract 75 mg, California poppy 20 mg + elemental Mg 75 mg (Sympathyl^®^)	Placebo	12 weeks	Hamilton Anxiety Scale Self-reported anxiety (100 mm VAS) Physician global impression	Total anxiety score: Significant decrease in both conditions. Effect larger in treatment group. Mean change from baseline between Day 0 & Day 90 significantly greater in treatment group (*x* = −10.6 (SD = 1.2)) vs. placebo (*x* = −8.9 (SD = 1.2); *p* = 0.005). Somatic score change from baseline: Treatment (*x* = −6.5 (SD = 0.7)) Placebo (*x* = −5.7 (SD = 0.7); *p* = 0.054). Self-rated anxiety VAS: Mean change from baseline between Day 0 & Day 90 significantly greater in treatment group (*x* = −38.5) vs. placebo (*x* = −29.2; *p* = 0.005). Physician global impression: benefit > risk rating significantly greater in treatment (90%) vs. placebo (80%; *p* = 0.0018).	+
Cazaubiel & Desor [58]	RCT	Mild anxiety (Hospital Depression & Anxiety Scale (HADS) score 4–12)	*N* = 80 (Treatment *n* = 40; Placebo *n* = 40)	26M:54F	Not reported	Fermented cow’s milk drink (100 mL) containing milk protein hydrolysate 222 mg + Mg 48 mg (Mg form unknown) + blackberry puree	Placebo	4 weeks	HADS Symptom Checklist Cohen Perceived Stress Scale Vitaliano Coping scale	No significant difference between treatment & placebo on study outcome measures. Post hoc analysis on restricted data (HADS anxiety subscale score 4–8, excluding scores ≥ 9) revealed significant decrease of 31% in treatment group (*n* = 15) vs. placebo (*n* = 16) on the anxiety sub-scale of the HADS (*p* < 0.05).	+ ^2^
Gendle et al. [76]	RCT	Subjective anxiety (Westside Test Anxiety Scale; normal anxiety; elevated normal anxiety; high anxiety; very high anxiety)	*N* = 122	31M:91F	*x* = 19.3 (SD = 1.17; 18–22)	Mg 300 mg as Mg citrate	Placebo (gelatin)	5 days	Spielberger State-Trait Anxiety Inventory	No significant difference between treatment and placebo on pre-exam anxiety rating.	x

^1^ Total Score > 20, with sum of 2 first items < 5 & score for item 6 (depressed mood) < 2; ^2^ Post hoc analyses; * No difference between treatments; Mg—Magnesium; mg—milligrams; VAS—visual analogue scale; + positive treatment effect; x—no treatment effect; RCT—randomised controlled trial; Hospital Anxiety & Depression Scale—HADS; SD—standard deviation.

**Table 2:** Summary of studies reporting the effects of Mg on subjective anxiety/stress in individuals reporting premenstrual syndrome symptoms.

Author	Study Design	Condition	Sample (*N*)	Age (year)	Treatment (s)	Control	Duration	Outcome Measure	Results	Effect Summary
Facchinetti et al. [88]	RCT Cross Placebo Cross	Premenstrual symptom complaints Moos Menstrual Distress Questionnaire (2 consecutive cycles (DSM-IIIR criteria))	*N* = 28	Placebo: *x* = 31.6 (SD = 5.9; 24–39); Treatment: *x* = 32.4 (SD = 6.2; 24–39)	Mg 360 mg as Mg pyrrolidone carboxylic acid	Placebo	2 months baseline + 4 menstrual cycles. Treatment: Mg x 2 2 cycles; placebo: placebo x 2 cycles + Mg x 2 cycles (intake during luteal phases only)	Moos Menstrual Distress Questionnaire (8 symptom categories: pain, inability to concentrate, autonomic reactions, water retention, negative affect, arousal, total score).	Mg significantly reduced negative affect ratings in the placebo crossover group (*x* = 0.51 (SD = 0.45)) vs. placebo intake (*x* = 0.76 (SD = 0.70); *p* < 0.05) & in the Mg treatment group after 2 (*x* = 0.44 (SD = 0.47)) & 4 (*x* = 0.45 (SD = 0.46)) cycles vs. baseline (*p* < 0.02).	+
Walker et al. [89]	R-Cross	Premenstrual symptom complaints Menstrual Health Questionnaire (MHQ; retrospective assessment of symptoms during last cycle)	*N* = 38	18–50 (71%–18–25; 7.9%–26–34; 13.2%–35–41; 7.9%–45–50)	Mg 200 mg as Mg oxide	Placebo (cellulose)	4 menstrual cycles (2 cycles per treatment)	22 item ordinal daily menstrual symptom diary (6 symptom categories: anxiety; cravings; hydration, depression, other, total)	No significant effect of treatment on anxiety related premenstrual syndrome symptoms.	x
De Souza et al. [67]	R-Cross	Premenstrual symptom complaints Menstrual Health Questionnaire (MHQ; retrospective assessment of previous month and baseline)	*N* = 44	*x* = 32	(i) Mg 200 mg; (ii) vit B6 50 mg; (iii) Mg 200 mg + vit B6 50 mg (as Mg oxide)	Placebo	5 consecutive menstrual cycles	30 item ordinal daily menstrual symptom diary (6 symptom categories: anxiety; cravings; hydration, depression, other, total)	No overall treatment effect. Predefined factorial treatment contrasts of adjusted mean scores showed a significant effect of Mg 200 mg + vit B_6_ 50 mg (*x* = 16.3) for reducing anxiety related premenstrual symptoms vs. baseline (*x* = 29.3) & placebo (*x* = 19.8; *p* = 0.04) for one menstrual cycle.	+ ^1^
Walker et al. [64]	R-Cross	Premenstrual symptom complaints Menstrual Health Questionnaire (MHQ; retrospective assessment of previous month and baseline)	*N* = 85	*x* = 35	(i) Mg 200 mg; (ii) Mg 350 mg; (iii) Mg 500 mg (all as Mg oxide)	Placebo (sorbitol 1305 mg)	2 menstrual cycles per condition	20 item ordinal daily menstrual symptom diary (6 symptom categories: anxiety; cravings; hydration, depression, other, total)	Significant reduction in anxiety-related premenstrual symptoms after 2 months placebo (sorbitol) intake (*x* = 1.7 (SD = 2)) vs. 200 mg (*x* = 3.6 (SD = 2)), 350 mg (*x* = 2.8 (SD = 2)) & 500 mg (*x* = 3.2 (SD = 2)) Mg treatments.	x
Khine et al. [63]	P Post-hoc R-Cross	Premenstrual complaints / Premenstrual Dysphoric Disorder (PMDD) Daily premenstrual symptoms VAS (3 months) & retrospective DSM-IV criteria for PMDD	*N* = 31 (PMDD *n* = 17; Placebo *n* = 14)	Control: *x* = 28.6 (SD = 6.4; 20–43); PMDD: *x* = 37.4 (SD = 4.4; 20–43)	Mg sulphate intravenous infusion 0.1mmol/kg body mass (4 h)	Premenstrual complaint-free controls	24 h post infusion	Spielberger State-Trait Anxiety Inventory Premenstrual Tension Scale (Subjective & Objective) 100 mm premenstrual symptom VAS	No significant mood changes in controls. Evidence of improved VAS mood ratings in initial 6 PMDD women after Mg infusion resulted in post hoc initiated RCT-cross with remaining 10 PMDD women receiving Mg & placebo infusion. Mg infusion subsequently demonstrated to have no mood improvement effects above placebo.	x
Quaranta et al. [85]	NR-Cross	Premenstrual symptom complaints Moos Modified Menstrual Distress Questionnaire (baseline score ≥ 25)	*N* = 38	x = 32.6 (SD = 8.0; 18–45)	Mg 250 mg (Mg form unknown)	None	3 menstrual cycles	Moos Modified Premenstrual Distress Questionnaire (including symptom categories: nervous tension, mood swings, irritability, anxiety). Monthly subjective PMS symptom diary	Moos Modified Menstrual Distress Questionnaire: Total score: Significant reduction after 3 months (*x* = 19.7 (SD = 7.6)) vs. screening visit (*x* = 30.5 (SD = 4.5); *p* < 0.001). Monthly subjective PMS symptom diary: Total score: Significant reduction at month 1 *(x* = 23.3 (SD = 10.6)), month 2 (*x* = 19.6 (SD = 7.8)), & month 3 (*x* = 17.9 (SD = 7.3)) with treatment vs. baseline months 1 (*x* = 31.8 (SD = 6.4)) & 2 (*x* = 31.3 (SD = 8.4); *p* < 0.001). PMS anxiety subscale: Significant decrease in anxiety subscale ratings at month 1 (*x* = 6.3), month 2 (*x* = 5.3), & month 3 (*x* = 5.0) with treatment vs. baseline (*x* = 8.4; *p* < 0.001).	+
Fathizadeh et al. [87]	RCT	Premenstrual symptom complaints Daily premenstrual symptoms record (2 months)	N= 116 (Treatments (i) *n* = 38; (ii) *n* = 41; Placebo *n* = 37)	Placebo: *x* = 28.03; Treatment (i): *x* = 28.71; Treatment; (ii): *x* = 30.02 (all 15–45)	(i) Mg 250 mg; (ii) Mg 250 mg + vit B_6_ 40 mg (Mg form unknown)	Placebo	2 months	Daily menstrual symptom diary (6 symptom categories: anxiety, cravings, hydration, depression, somatic, total)	Significant reduction in total PMS symptoms in all conditions. Mg + vit B6 resulted in greatest reduction (*p* < 0.05). Significant main effect of treatments on change from baseline anxiety ratings (Mg + vit B_6_: *x* = −22.61 (SD= 20.36); Mg: *x* = −12.14 (SD = 26.14); placebo: *x* = 0 (SD = 20.41); *p* < 0.001). However, no between treatment planned contrasts or post-hoc tests reported.	+?

^1^ Post hoc analyses; Mg—Magnesium; mg—milligrams; PMDD—Premenstrual Dysphoric Disorder; PMS—premenstrual syndrome; VAS—visual analogue scale; MHQ—Menstrual Health Questionnaire; + positive treatment effect; x no treatment effect; ?—doubts about outcome; RCT—randomised controlled trial; P—parallel groups; R-Cross—randomised crossover; NR-Cross—non-randomised crossover; B_6_—vitamin B_6_; SD—standard deviation.

**Table 3:** Summary of studies reporting the effects of Mg on subjective anxiety/stress in postpartum women.

Author	Study Design	Condition	Sample (*N*)	Sex	Age (year)	Treatment (s)	Control	Duration	Outcome Measure	Results	Effect Summary
Fard et al. [68]	RCT	Postpartum ≤48 h	*N* = 95 (Treatments: (i) *n* = 31; (ii) *n* = 31; Placebo: *n* = 33;	F	Treatments: (i) *x* = 29.4 (SD = 5.4); (ii) *x* = 26.4 (SD = 4.8); Placebo *x* = 27.6 (SD = 5.1)	(i) Zinc sulphate 27 mg (11 mg elemental zinc); (ii) Mg sulphate 320 mg (64.6 elemental Mg)	Placebo (lactose, starch, cellulose, Mg stearate)	8 weeks	Spielberger State-Trait Anxiety Inventory	No significant differences between treatments	x

Mg—Magnesium; mg—milligrams; + positive treatment effect; − negative treatment effect; x no treatment effect; RCT—randomised controlled trial.

**Table 4:** Summary of studies reporting the effects of Mg on subjective anxiety/stress in individuals with mild to moderate hypertension.

Author	Study Design	Condition	Sample (*N*)	Sex	Age (year)	Treatment (s)	Control	Duration	Outcome Measure	Results	Effect Summary
Borrello et al. [70]	RCT	**Mild hypertension** (Diastolic BP > 90 mmHg or Systolic BP > 140 mmHg)	*N* = 83 (Treatment *n* = 42; Placebo *n* = 41)	30M:53F	Placebo: *x* = 49; Treatment: *x* = 51	Mg oxide 200 mg	Placebo	12 weeks	44 item Quality of Life Likert questionnaire (subscales: emotional behaviour & concerns about the future)	Significantly higher total quality of life rating after 12 weeks treatment (*x* = 67.58 (SD = 5)) vs. baseline (*x* = 73.58 (SD = 6)) & placebo (*x* = 73.23 (SD = 8); *p* < 0.05).	+
Walker et al. [64]	RCT	**Mild hypertension** (Diastolic BP 85–100 mmHg)	*N* = 36 (9 per condition)	18M:18F	Placebo: *x* = 49; Treatment (i): *x* = 53.2; Treatment (ii): *x* = 53; Treatment (iii): *x* = 48.8	(i) Mg amino acid chelate (600 mg elemental Mg/day); (ii) Hawthorn extract 500 mg; (iii) (i) + (ii) combined	Placebo (cellulose)	10 weeks	Subjective well-being questionnaire (subscales: vitality, anxiety & depression)	No significant effects on subjective well-being.	x

Mg—Magnesium; mg—milligrams; + positive treatment effect; − negative treatment effect; x no treatment effect; RCT—randomised controlled trial.

## Appendix A

Grey Literature Search Resources

PsychExtra: www.apa.org/pubs/databases/psycextra/

Open Grey: www.opengrey.eu/

Google Scholar (first 1000 returns per search): https://scholar.google.co.uk/

TRIP database: https://www.tripdatabase.com/

Information for Practice: http://ifp.nyu.edu/

Grey Literature Report: http://www.greylit.org/

Latin American Open Archives Portal: http://lanic.utexas.edu/project/laoap/

British Library EThOS eThesis online service: http://ethos.bl.uk/Home.do

National Institute for Health Research (INVOLVE): http://www.invo.org.uk/

The OAlster Database: www.oclc.org/oaister.en.html

Health Management Information Consortium: www.lshtm.ac.uk/library/resources/databases/info_hmic.html

UK Department of Health: www.gov.uk/government/organisations/department-of-health

Centers for Disease Control and Prevention: www.cdc.gov

National Institute of Health: www.nih.gov

World Health Organization: www.who.int/en/

European Food Safety Authority: www.efsa.europa.eu
